# Supplementary material for: Inhibiting Monocyte Recruitment to Prevent the Pro-Tumoral Activity of Tumor-Associated Macrophages in Chondrosarcoma
Source: Cells. 2020 Apr 24;9(4):1062. doi: 10.3390/cells9041062 (PMC7226304; doi:10.3390/cells9041062)
Supplement: Supplementary file 1 [file cells-09-01062-s001.pdf]

Supplementary Data

.

Pearson's Correlation coefficients

	CD68 Average	CD68 Score	CD163 Average	CD163 Score	iMVD Average	iMVD Score	PFS
**CD68 Average (n.18)**	1	0,835	0,855	0,815	0,834	0,746	0.013
**CD68 Score (n.18)**	0,835	1	0,897	0,911	0,752	0,657	0,044
**CD163 Average (n.18)**	0,855	0,897	1	0,959	0,929	0,882	-0,485
**CD163 Score (n.18)**	0,815	0,911	0,959	1	0,854	0,844	-0,285
**iMVD Average (n.18)**	0,834	0,752	0,929	0,854	1	0,962	-0,573
**iMVD Score (n.18)**	0,746	0,657	0,882	0,844	0,962	1	-0,426
**PFS (n.8)**	0.013	0,044	-0,485	-0,285	-0,573	-0,426	1

Figure S1. Relationship between CD68 and CD163 and CD31 expression levels in tumor tissues and progression free survival of CHS patients.

Values of Pearson's correlation coefficient referred to the correlation between $CD68^{+}$, $CD163^{+}$ and intratumor microvessel density(iMVD) CD31 averages and scores, and progression free survival,were assessed by using the SPSS 20.0 software. In parentheses is the number of analyzed cases.

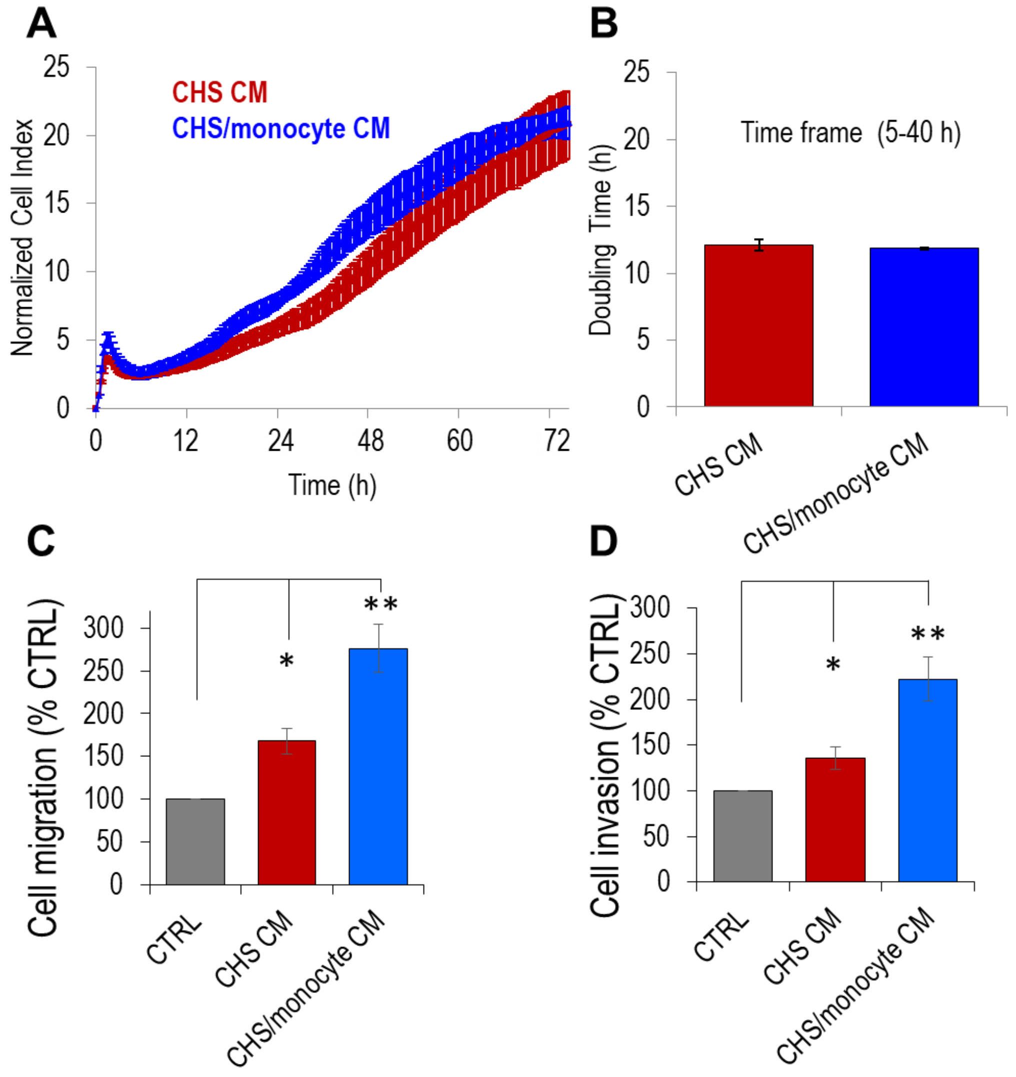

Figure S2. Proliferation and migration rate of CHS cells.

Proliferation rate of primary CHS cells derived from the tumor tissue #9 assessed by monitoring impedance by RTCA xCELLigence Thecnology. **A**. Primary CHS cells were seeded in E-plates in CM from CHS cells or CM from human monocytes pre-co-cultured with CHS cells. In all cases, serum was added to a 5% final concentration. **B**. Doubling times were calculated from the cell growth curves, during exponential growth (time frame: 5-40 h). Data represent mean ± SD from a quadruplicate experiment representative of 3 replicates. **C-D.** Cell migration **(C)** and invasion **(D)** of CHS cells in Boyden chambers. CHS cells were allowed to migrate (**C**) or invade matrigel (**D**) toward serum-free medium (CTRL), CM of human monocytes recovered after monocyte/CHS co-cultures or control CM from CHS cells alone. In all cases, FBS was added to a 5% final concentration in the lower compartment of Boyden chambers. For quantitative analysis, values were reported as percentage of the CTRL, considered as 100%. Data are the means ± SD of three independent experiments, performed in triplicate. Statistical significance with *$p < 0.005$ and **$p<0.001$.

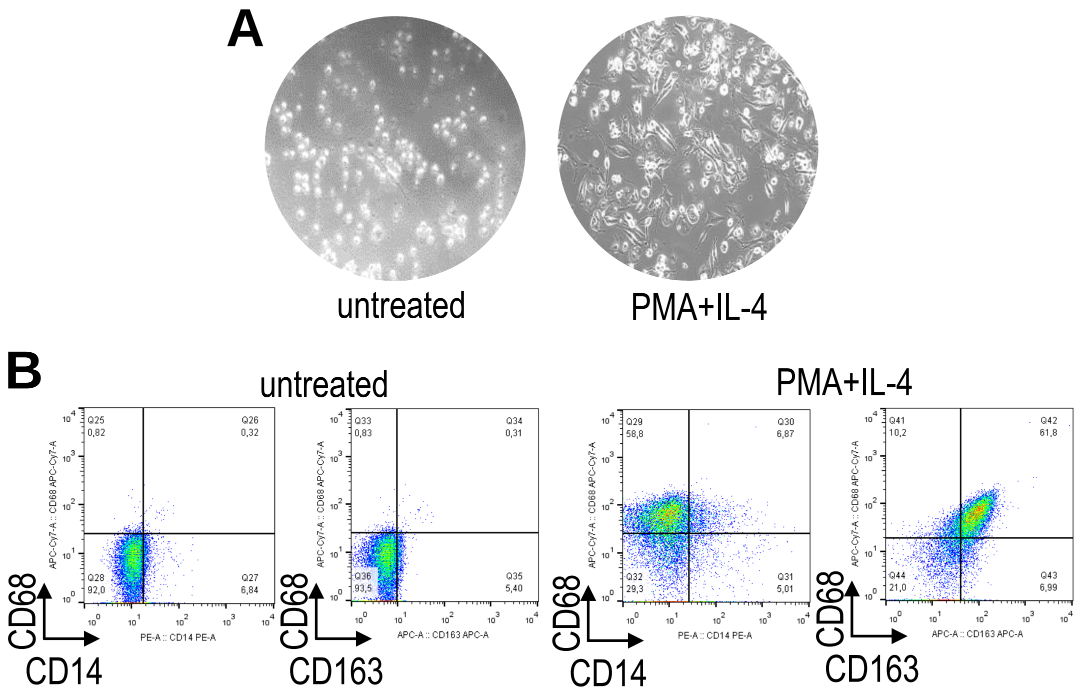

C

untreated			PMA+IL-4		
CD14 (%)	CD68 (%)	CD163 (%)	CD14 (%)	CD68 (%)	CD163 (%)
7.16	1.14	5.71	11.88	68.84	68.79

Figure S3. Flow Cytometric analysis of CD68 and CD163 expression in unreated and PMA/IL-4-treated THP-1 cells. Untreated and PMA/IL-4-treated THP-1 cells **(A)** analyzed for CD14, CD68 and CD163 expression by flow cytometry **(B).** Percent of cells expressing CD14, CD68 and CD163 in untreated THP-1 cells and in cells treated with PMA/IL-4.
